# Supplementary material for: Molecular xenomonitoring of diurnally subperiodic Wuchereria bancrofti infection in Aedes (Downsiomyia) niveus (Ludlow, 1903) after nine rounds of Mass Drug Administration in Nancowry Islands, Andaman and Nicobar Islands, India
Source: PLoS Negl Trop Dis. 2020 Oct 23;14(10):e0008763. doi: 10.1371/journal.pntd.0008763 (PMC7641468; doi:10.1371/journal.pntd.0008763)
Supplement: Attachment — Submitted filename: Reviewer 393.pdf [file pntd.0008763.s001.pdf]

PNTD-D-19-01106

<u>**Reviewer-393**</u>

The present study was carried out to evaluate the impact of nine rounds of Mass Drug Administration (MDA) of DEC/Alb (or of DEC-fortified salt as mentioned in the Introduction, Line 110? And in the abstract as well? This is not clear) in four islands endemic for diurnal subperiodic *Wuchereria bancrofi* infection (Nancowry, Nicobar district, India). The day biting and outdoor resting mosquito, *Downsiomyia nivea* is the filaria vector in these islands. Molecular xenomonitoring (MX) was proposed to complement unpublished data on persisting human microfilaremia and for monitoring the outcome of MDA in 25 villages of these islands. For this, four mosquito collection methods were compared: BioGents Sentinel (BGS) traps, gravid traps, human baited double bednet traps and man landing collections (MLCs). Only MLCs yielded enough day biting *Do. nivea* for MX.

The objectives of the study could have been better defined. As presented, however, they served the purpose of the study. The methodology used for mosquito collection and for MX is relevant to the described objectives of the study.

After nine rounds of MDA, vector infection was detected in all four islands. PoolScreen estimate of mosquito infection with parasite DNA was much above the critical value (0.25%) below which filarial transmission cannot be sustained. The authors reported that MDA should be continued in these villages. The authors discussed the fact that people inhabiting these islands were still at risk of acquiring filaria infection after 12 rounds of MDA (anonymous publication, 2011). Furthermore, an unpublished study by Shriram et al., 20191) revealed that one year after the mass distribution DEC-fortified salt, mf prevalence declined to less than 1%. It would have been interesting to compare mosquito infection by MX between nine and 12 rounds of MDA, and after the distribution of DEC-fortified salt. The present data seems somewhat lagging.

The manuscript contains grammatical errors that need to be addressed. English writing needs to be improved as well.

Issues raised below point out to specific statements in the Word file of the manuscript with page and line numbering.

Major issues

1- The sequence of data reported in the Results section is not correct. Table 1 reports details of trapping devices for *Do. nivea*. Table 2 reports means of numbers (where are the numbers?) of collected mosquitoes according to mosquito species, trapping device and location, regardless of island. Numbers of the different mosquito species collected are presented elsewhere in a Figure. Table 3 presents data for means (where are the numbers?) of three of the mosquito species collected (why these species in particular?) according to island and village.

A logical sequence would have been to present the data as follows: Mosquito species collected, their numbers (the Figure) and mean per trap (Table 2 would become Table 1) regardless of location. Then may be data in Table 3 (to be renamed Table 2) which reports means of means (!!!) for three mosquito species (provided the choice of these particular species is stated) according to island and village. Then concentrate on *Do. nivea* which is the filaria vector in selected study sites. In this prospect, Table 1 (to be renamed Table 3) is presented, then Table 4, and finally Table 5. A propos, potential filaria mosquito vectors in inland India and collected herein could not be tested for filarial infection?

2- Writing is confused. On many occasions one does not know whether numbers presented are for both male and female mosquitoes or for females solely.
3- **P4, 79-80."** MDA with diethylcarbamazine citrate (DEC) and albendazole (alb) is the strategy to interrupt transmission". The abstract (Line 35) mentions that MDA was based on the administration of fortified DEC salt.
4- **P5, L110-111:** "The present study was carried out to assess vector infection to evaluate the impact of nine rounds of MDA". With DEC/Alb? With DEC-fortified salt? The national program was initiated in the islands in 2004. When was this study carried out? Please specify.
5- **P6, L119-120:** a map would be useful for locating these islands.
6- **P7, L142-144:** "Assuming an expected microfilaraemia prevalence of 1%, the sample size derived was 2140 mosquitoes for the four islands". Considering the study area (638.7 km^2), the number of villages (25) and the population size (10,634), this sample size seems extremely small to me. How was it calculated?
7- The Results section reports numbers without indicating their significance in relation to the objectives of the study. What do these numbers indicate?
8- The discussion section writes: "None of the trapping devices (BGS, gravid and HBDN) was productive for the forest-dwelling vector mosquito. The females frequent the human dwellings during the day time besides contacting the people visiting the forest. Due to this unique feeding behaviour of this vector mosquito, trap collections were not productive **(P23, L366-370)**". Such interpretation is not convincing. BGS-Lure and HBDN set at day time signaled to host-seeking mosquitoes the presence of a potential human bloodmeal. Such trapping methods attracted predominantly *Aedes albopictus* and *Aedes aegypti* and very few *Do. nivea*. Was the category of lure adapted to *Do. nivea*?

   Also, **P24, L379-380** reports that two layers of nets proper to HBDN could have acted as a physical barrier to host-seeking mosquitoes. By reducing the human signal?

   Gravid mosquitoes do not seek blood; but seek a suitable oviposition site, in occurrence the prepared media in set gravid traps.

   Was there enough *Do. nivea* around at the time of collection? The study does not specify the time period and weather conditions at which collections were carried out.
9- Discussion on relative efficacy of BGS and CDC traps for capturing host-seeking mosquitoes **(P24, L380-382)** is irrelevant to the present study and does not lead to a recommendation.
10- Table 5 shows microfilaria prevalence in these villages; but no reference is made to it in the results section. Villages with no microfilaria, contained infected mosquitoes, with PoolScreen estimation sometimes exceeding the 0.25% provisional threshold. Also, the authors failed to

report that the upper confidence limit exceeded such threshold in all screened villages. This should be commented on in the discussion.

Minor issues

Title: *Downsiomyia nivea* (L). L. traditionally refers to Linnaeus. You should rather write *Downsiomyia nivea* (Ludlow, 1903).

P5, L96-97. "Annual rounds of MDA were continued; 9 rounds of MDA had been accomplished so far". Please specify the year instead of so far.

P5, L104-108: |The levels of vector infection, assessed based on the statistically robust method and molecular assay and its relationship with human infection are not known for this form of filariasis in Nancowry, to understand the scope of this strategy in evaluating MDA for decision making and post MDA surveillance". Should be more clearly stated.

P6, L124: "through A & N Administration". A? N?

P6, L125-126: "During post-tsunami period". When was that?

P6, L132-133: "man landing collections were carried out in all the 25 villages in four islands". Is Chowra the fourth island?

P8, L165-167: The materials and methods describe the methodology used solely. No results in this section.

"Overall, in 21 villages, a total of 235 trap collections spread over 47 trap days involving a total of 2350 hours, were completed (Table 1), 65 trap collections in each of domestic and peri-domestic settings, and 105 in the sylvan locations". This should be in the results section. Same comment regarding gravid trap and HBDNT collections. **Lines 180-182 and Lines 210-211** should be moved to the Results section as well.

P8, L178-180: "A total of 153 trap collections spread over 51 days were made, spending 918 hours (Table 1). These traps were set on three consecutive days for each of the settings at monthly intervals.". Dates for mosquito collection are not specified, season? Month? Monthly? Consecutive days? This is unclear. **Lines 166-167** report that BGS traps were hung over 47 days. Month? Consecutive days?

P8, L185: "Mosquitoes collected were identified". Please mention reference used.

P9, L 200-203: "After every 10 minutes, the insect collector would approach and catch mosquitoes resting in the outer net. Then, the external mosquito net was rolled up and all the trapped mosquitoes were captured with oral aspirators by insect collector". I understand that mosquitoes were collected at 10 minutes intervals (from 6 AM to 1 PM? Or for how long?). After 1 PM the outer net was rolled up and trapped mosquitoes collected?

P11, L210-212: "Only 393 mosquitoes including 44 *Do*. *nivea* (11.2%) were collected by all the three trapping devices". Should be moved to the results section.

P11, L223: "*Do. nivea* mosquitoes were pooled with 10 each". Pools of 10 *Do. Nivea* were those collected by MLCs? Or this included those collected by other trapping devices? Please specify.

P12, L 240-241: "and water controls." Specify water negative control.

P14, L273: "BGS trap collections yielded 53 male and 220 female mosquitoes". you could refer to the Figure here. Refer to the figure, as well, for the gravid trap and HBDNT paragraphs.

P14, L273-274: "*BG-Sentinel Trap*: BGS trap collections yielded 53 male and 220 female mosquitoes. Per trap collection was 0.9 females". Specify that these numbers are overall the 21 villages, with less than 1 female per trap (range …. - …..).

P14, L274-275: "Six species of mosquitoes were trapped and *Ae*. *albopictus* was relatively more in number (n=88, 40.0%) followed by *Ae*. *aegypti* (n=72, 32.7%). Are these mosquitoes of both sexes or females only? Please specify.

P14, L278-279: "Collections from the three mosquito collection sites, were comparable with reference to the number of *Ae*. *albopictus* and *Ae*. *aegypti* collected". This is unclear. Please explain.

P14, L280-282:" The mean density of vector mosquito was 0.2 per trap from sylvatic and 0.03 per trap from peri-domiciliary mosquito collection sites (Table 2)". If you are referring to *Do. Nivea*, such numbers are 0.02 and 0.07 (Table 2).

P16, L287-288: "The per trap density of *Ae*. *aegypti*, *Ae*. *albopictus* and *Do*. *nivea* in the three mosquito collection sites across the 21 villages is given in Table 3". I guess by collection site you mean "island" and not "domestic, peridomestic and sylvatic". Please clarify.

P16, L288-289: "The number of *Do*. *nivea* per trap in these three ranged from 0 to 0.2, 0 to 0.4 and 0 to 1.2 respectively". By "these three" are you referring to islands? Or to domestic, peridomestic and sylvatic? In either case, by looking at Table 3, I could not verify these numbers. Again, means are so small (less than one), that the statistics does not add much.

P18, L296-297: "From 510 gravid trap collections, a total of 73 female mosquitoes belonging to 7 species were collected. Mean per trap density was < 1". The figure shows that 80 females were recovered from gravid traps. What was the range per trap?

P18, L297-300: "The mosquito species recorded in gravid traps in their order of abundance were *Ae*. *albopictus* (n=30, 41.0%), *Ae*. *aegypti* (n=25, 34.3%), *Do*. *nivea* (n=9, 12.3%), *Ae*. *malayensis* (n=5, 6.9%),

Cx. *quinquefasciatus* (n=2, 2.7%), *Ae*. *edwardsi* (n= 1, 1.4%), and *Ar*. *subalbatus* (n=1, 1.4%) from the three settings (Figure)". These numbers are different from the ones shown on the Figure.

P18, L300-301: "The number of *Do*. *nivea* collected were too small to make any further analysis". However, number per gravid trap of females of this species is presented in Table 2.

P18, L303-306: "A total of 51 HBDNT collections were completed and 100 female mosquitoes belonging to 6 species viz. *Ae*. *aegypti* (n=44, 44.0%), *Ae*. *albopictus* (n=34, 34.0%). *Do*. *nivea* (11.0%), *Ae*. *malayensis* (2.0%), *Cx*. *quinquefasciatus* (8.0%), and *Ar*. *subalbatus* (1.0%)". No need to repeat the numbers here as they are shown in the Figure. Just refer to the Figure.

P18, L315-316: "A total of 2170 female mosquitoes of *Do*. *nivea* were collected". Would have been nice to report this number in the Figure, as well as the number of other mosquito species recovered by this method.

P18, L317-318: "The density of man landing mosquitoes varied between the sampling sites (ANOVA *df* =24, *F*=3.015, p=0.05)". A non-parametric test seemed more appropriate.

P19, L318-319: "The mean man landing density ranged from 37.75 (Teressa) and 96.94 (Kamorta) per person per collection". If man landing rate is meant, this number is 44.58 (Table 4).

P21, L329-330: "215 pools were processed by RT-PCR". This totalizes 2,150 females according to Table 5. Are these mosquitoes from MLC's only?

P21, L331-332: Please add % after each percentage reported.

P21, L334-336: "The pool screening calculation indicated a maximum likelihood estimate (MLE) of infection of 0.71% (95 % CI: 0.25–1.86%) in Chowra, followed by 0.37% (95 % CI: 0.07–1.22%). PoolScreen mosquito infection amounted 0.77% in Chowra. It seems the rest of the sentence is missing.

P23, L 353: "A&N islands". No abbreviation please.

P23, L354-357: "*Mf* survey is operationally feasible in this situation since only day blood samples are needed. However, most of the Nicobarese visit the forest to collect forest products and their availability for blood sampling during the day time remain uncertain. In such situations, xenomonitoring can be a supplementary method of choice". Important information, nice to be pointed out in the Introduction.

P26, L432: "there is a need to identify alternative methods of vector sampling". Any suggestion for an alternative method?

Abstract

Prevalence of vector infection prevalence: delete the second prevalence.

The conclusion in the abstract needs to point out the significance of PoolScreen estimated mosquito infection rate after 9 rounds of MDA.

Keywords: Andaman-Nicobar-Is. Is should be islands?

Summary

"Wuchereria bancrofti, spread across 73 countries". Rather 83 countries (See:World Health Organization, 2006. Global Program to Eliminate Lymphatic Filariasis. *Wkly Epidemiol Rec 81:* 221-232).

"viz". Is this an abbreviation? For what?

"Persistence of infection was evident despite 12 rounds of MDA. We studied filarial infection in the vector mosquito". In the present study, mosquito infection was estimated after 5 rounds of MDA (with what?).

"the infection in *Do*. *nivea* was 0.43 which is above the threshold of 0.25". 0.25 is a provisional threshold.

"we compared the results of xenomonitoring with community microfilaria prevalence (*mf*)". Mf prevalence is not mentioned in the materials and methods, neither in the abstract. What was the output of this comparison?

Table 1

Too many repetitions, too many numbers

No. of traps might be confusing at first sight (per day? Total?) Trap/day in that column is clearer.

Total duration (N0. Traps x average time). Easy to calculate, delete column.

Average collection time did not vary in trap or day? If not, no need to specify an average.

References in @ and # can be mentioned in the Materials and Methods, therefore no need to include it in the Table. Instead I would use the various symbols (for example, $GR^1$for cumin seed infusion, GR^2 for cashew leaves. Lure and human baits may as well be referred to in the footnote and the whole attractant column deleted.

Table 2. The title is confusing. What does "per trapping sampling period" mean? Also, I understand from the title that numbers reported are for females only. However, the text (lines 273-282) report the number of mosquitoes regardless of sex.

The table compares means between collection sites for *Do. nivea* only and reports the statistics. Numbers are so small (less than one female) that the comparison is meaningless. I would delete the statistics.

Table 3. Why does Table 2 report data for seven mosquito species regardless of location, whereas Table 3 presents data for three only of these species according to location (island and village)?

Instead of reporting means for each village (with no number shown), this could be replaced by numbers for each village, then mean ± SD in subtotal (to be renamed sub-mean?).

Table 4. Titles of columns need clarification. No. MLC is the number of volunteers? What do you mean by sampling site? How were Total man/hour and man landing rate calculated?
What is meant by per man hour density and how was it calculated? Man hour density is made clear in the Discussion section (L372-373); but this and other calculations should be mentioned in the Materials and Methods.

Table 5. The title makes no mention of *Do. nivea*.
I would title Table 5 something as follows:
PoolScreen estimation of *W. bancrofti* infection in *Do. nivea* mosquitoes after nine rounds of MDA with ….. , in Nancowry islands, India, 2014-2015.
S. No. refers to what? I would replace it by "Village" or Village No.
Rename column 6 into: No. (%) positive pools

Pool positivity and estimated prevalence of filarial DNA in the Nancowry islands, 2014-15

$ refers to: Shriram et al. unpublished pool size =10, and not to microfilaremia by site.

Figure Seems to be misplaced**.** MLC'S are not represented in the Figure although it yielded the highest number of *Do. nivea* compared to other trapping methods.
