# Supplementary material for: Molecular xenomonitoring of diurnally subperiodic Wuchereria bancrofti infection in Aedes (Downsiomyia) niveus (Ludlow, 1903) after nine rounds of Mass Drug Administration in Nancowry Islands, Andaman and Nicobar Islands, India
Source: PLoS Negl Trop Dis. 2020 Oct 23;14(10):e0008763. doi: 10.1371/journal.pntd.0008763 (PMC7641468; doi:10.1371/journal.pntd.0008763)
Supplement: Attachment — Submitted filename: Response_outstanding issues.docx [file pntd.0008763.s005.docx]

**Molecular xenomonitoring of diurnally subperiodic *Wuchereria* *bancrofti* infection in *Aedes* (*Downsiomyia*) *niveus* (Ludlow, 1903) after nine rounds of Mass Drug Administration in Nancowry islands, Andaman and Nicobar Islands, India" (#PNTD-D-19-01106R1)**

1. *Please include a description of the timing of the MLCs per village. For example, an additional column in Table 2 to say which month(s) the MLCs occurred in each village.*

*Response*: Description of the timing of MLCs conducted in each village is given in the revised manuscript (Page 8; Line:171-172). As suggested a column has been added in the Table 2, showing the period of MLC.

2. *Please explain why MLCs were only conducted in sylvan areas*

*Response*: The native Nicobarese spend considerable part of the day working in the forests/coconut plantations for harvesting copra and miscellaneous food articles. *Ae*. *niveus* is a sylvatic mosquito, breeding in tree holes in the forest and resting outdoors. It is learnt from the local tribal community that they are bitten by mosquitoes when they frequent the forests. Anticipating that the vector species is an opportunistic biter on the people engaged in forest, MLCS were conducted in the sylvan areas only. This explanation is given in the revised manuscript (Page12; 262-268).

3. *There could have been bias in assessing how effective the traps were compared to MLCs because the traps were mostly set during different months of the year (described in the text as not corresponding to the peak times for Ae. niveus) and also at different times of the day (also described in the text as not corresponding to the peak biting times of Ae. niveus). This should be mentioned/explored in the discussion*.

*Response*: Agreed. Collections were conducted at different periods of time depending on the accessibility to the study sites located in different islands. Comparison of results of different collections could have the bias, which has been mentioned in the discussion section of the revised manuscript (Page 18. Lines 417-419).

4. *Mosquitoes were clearly pooled by location, but were they also pooled based on collection day*?

*Response*: Mosquitoes collected by MLCs in a given location and collection day were pooled separately. Each pool of mosquitoes representing the location and date of collection was considered as a sample to extract and assay for detecting filarial infection. This has been mentioned under methodology section of the revised manuscript (Page 13; Line 278-281)

5. *In line 422, the authors mention that Cx. quinquefasciatus were not analysed because they are day biting. Especially in this scenario where W. bancrofti is diurnally sub-periodic, it would stand to reason that day-biting Culex could also be an important vector. The authors should mention why this is not apparently the case--has it already been studies on these islands?*

*Response*: *Cx*. *quinquefasciatus*, the omnipresent and ubiquitous vector of nocturnal periodic form of *Wuchereria* *bancrofti* was less abundant (1.7% of the total mosquitoes collected) in our earlier study in the sites selected for the study. None of the *Cx*. *quinquefasciatus*, dissected for filarial infection was found positive [Shriram et al. 2005. Diurnal pattern of human-biting activity and transmission of sub-periodic *Wuchereria* *bancrofti* (filariidea: Dipetalonematidae) by *Ochlerotatus* *niveus* (Diptera: Culicidae) on the Andaman and Nicobar Islands of India. Am. J. Trop. Med. Hyg., 72(3), 2005, pp. 273–277]. This aspect has been incorporated in the discussion. In the present study, we could collect only 12 female mosquitoes of *Cx*. *quinquefasciatus* from gravid traps and HBDNT collections and hence not included for molecular assay. This explanation has been included in the revised manuscript under discussion (Page.19; Line: 448-452)

6. *Lines 432-433 specifically mention that 2 islands that had received DEC salt achieved total disruption of transmission. Did the MX data arising from this study corroborate this finding?*

*Response*: MX was not carried out after DEC salt distribution and the impact was assessed only from infection in human. However, MX can be considered in the islands which are currently under DEC salt intervention. This is mentioned in the revised manuscript (Page 20; Line:464-466).

7. Figure 3 (which is currently mislabelled as Figure 1): please adjust the y-axis to better accommodate the 2170 Ae. niveus captured by MLC; as it appears now, it is confusing to have 2 separate y-axes. To avoid loss of resolution when visualizing the other values, the authors may consider adding a 'break' on the y-axis to demonstrate that the uppermost value is not in scale with the others.

*Response*: Agreed that the Figure 3 was incorrectly labelled as 1 in the graph and has been corrected in the revised manuscript, Also the graph has been revised for better visualization.

8. *Please edit the full manuscript for English grammar and readability.*

*Response*: As suggested the full manuscript has been edited for English grammar and readability

In addition to the above, we have rectified the errors in the calculation of man landing rate and man biting rate (Table 2). Correspondingly, we have reframed the sentences in the results section (Page 16; Lines 356-359)
