# Supplementary material for: Molecular xenomonitoring of diurnally subperiodic Wuchereria bancrofti infection in Aedes (Downsiomyia) niveus (Ludlow, 1903) after nine rounds of Mass Drug Administration in Nancowry Islands, Andaman and Nicobar Islands, India
Source: PLoS Negl Trop Dis. 2020 Oct 23;14(10):e0008763. doi: 10.1371/journal.pntd.0008763 (PMC7641468; doi:10.1371/journal.pntd.0008763)
Supplement: Attachment — Submitted filename: Reviewer Comments.docx [file pntd.0008763.s002.docx]

**Summary**

Molecular xenomonitoring is a surveillance strategy to detect circulating filarial parasites in an endemic population which is either under intervention or post-intervention. It is an important surveillance tool when human sample collection is difficult. This manuscript provided data from an endemic area where parasite load is high even after many rounds of MDA. The parasite is diurnal in that area where most of the people are outside of their house so blood collection for diagnosis is difficult. Also they undertook many sampling strategies for the specific vector which will be an important add to the scientific literature and benefit scientists and researchers of similar interest. However, the manuscript has failed to clearly portrait its objective. Their assumption for sample collection is incorrect and lacks proper analysis. Some of the analysis lacks enough data. Description could be made in a better way as the article needed to be improved by rewording.

**Specific Comments**

**Abstract**

Background

Page 2, Line 30: How many rounds of MDA, 12 or nine?

**Method**

Line 38: Not all the collection methods were compared.

**Results**

Line 42: So mosquitoes from collected only from MLCs were analysed for the infection?

**Introduction**

Page 4, Line 72: Correct the information according to the reference. Also, is there any update epidemiology available?

Line 72: Replace persons by people

Line 77: Replace worldwide by global

Line 79-80: Are these two drugs used simultaneously?

Line 82: Latin name at the beginning of the sentence has to be full.

Page 5, Line 110: So DEC-fortified salts are also implemented, or it is the regular mode of intervention? It came all of a sudden. Tell something about the implementation before. What are you evaluating with xenomonitoring, MDA or DEC-salt or both?

Line 111: Add ‘by xenomonitoring of *Do. nivea*’ at the end of the sentence?

Line 112: Sampling method, placement, time and selection of region is very crucial for xenomonitoring. Perhaps add this in the introduction. As this manuscript indicated a successful sampling technique like MLC after comparing a number of methods.

**Materials & Method**

Page 6, Line 121: Reference needed.

Line 125: Mention the tsunami year.

Page 7, Line 142: Explain why the expected prevalence is 1% when it was already more than 1% in the islands.

Line 150: The trapping sites mentioned here are already mentioned the earlier paragraph. Again how many islands actually? Previously you have mentioned three out of four but in here it is three out of five?

Page 9, Line 186: This is totally unnecessary. Desiccants keep the mosquitoes good enough for PCR.

Page 11, Line 214: What is the timeline of the study?

Line 218: You have selected volunteers from the endemic region. Did you check if the volunteer already have the infection? How many volunteers have been used and how many MLCs were conducted? What is the follow-up process of the volunteers? Any benefit given to them?

Page 12, Data analysis, Line 253-255: Have to be careful with count data. Table 2 indicates that the data are over dispersed. Mean is not the proper way to compare between locations or traps. How did you measure the densities? Need better comparison tool like risk ratio for collection location. How did you calculate man landing rate?

Additional Comment: Can you provide a map of the islands with collection sites according to methods?

**Results**

Page 14: Line 273: Use the term ‘BGS’ uniformly

Line 281: Sometime mean and standard deviation is used and sometime not used. Be uniform throughout the document.

Page 15, Line 285: What are these?

Page 17, Table 3: The sample size is too low for any kind of comparison. Table 3 should be taken out.

Page 18, Line 300: Figure number missing

Line 298-300 and 304-306: The list of mosquitoes are not required. Just mentioned one or two dominant species collected from these traps.

Line 306-308: Re-write the sentence like: The mean number of mosquitoes collected per trap ranged between 0.07 to 0.38 in collection sites.

Line 309: Provide a figure number even if there is no more figure. DBNT or HBDNT?

Line 317: Why it is density?

Line 318-323: Be uniform providing mean and standard deviation

319: ‘96.94’ is it Kamorta or Nancowry?

Page 20, Table 4: Calculation of man landing rate and per man hour density is not clear. Provide description in the data analysis.

Page 21, Line 331: Give % after 7.6

Line 332: Same as previous

Line 337: Font not same, give 5 after 0.43

**Discussion**

Page 23, Line 352: Any possible reason behind the failure of so many MDA rounds?

Line 359: What is XM?

Line 359-361: Proper message from this sentence is missing. Mf prevalence >1% would provide better yield for surveillance by methods like xenomonitoring when it is difficult sample human blood.

Line 367: Are these supposed to be productive when the mosquito is forest dwelling?

Line 368: Anything missing from this sentence?

Line 370: Delete ‘to’

Page 24, Line 372-383: Re-write the whole paragraph. Surprised to see 10 per man hour rate in discussion when not mentioned the results. Why do a sample of 20 is required? Perhaps some more interesting discussion for success of MLC over HBDNT? What is the link of discussing on BGS here with this study?

Line 385-394: What is the context of discussing Ae. polynesiensis and BGS? Delete this paragraph or reword.

Page 25, Line 396-398: Merge the two sentences and a better rephrasing needed.

Line Line 398-400: This is an important finding supporting molecular xenomonitoring as a surveillance strategy for filariasis. Perhaps add some more evidence here. Bring the next paragraph here.

Line 406-411: There is repetition from the expression of present study outcome, merge the two sentences.

Page 26, Line 426: Give evidences from other countries

**Conclusion**

Page 26, Line 431: There are already many methods alternative to MLC but it’s just the species specific in this case.

Line 432-434: Not necessary.

Line 434-436: As commented before, it is not clear under what circumstance MX is potential. Is it for MDA, DEC-salt or both?

**References**

Check with the journal’s format guidelines. Doesn’t look like the style is followed properly.
