# Supplementary material for: Molecular xenomonitoring of diurnally subperiodic Wuchereria bancrofti infection in Aedes (Downsiomyia) niveus (Ludlow, 1903) after nine rounds of Mass Drug Administration in Nancowry Islands, Andaman and Nicobar Islands, India
Source: PLoS Negl Trop Dis. 2020 Oct 23;14(10):e0008763. doi: 10.1371/journal.pntd.0008763 (PMC7641468; doi:10.1371/journal.pntd.0008763)
Supplement: Attachment — Submitted filename: Reviewer Comments.docx [file pntd.0008763.s004.docx]

**Molecular xenomonitoring of diurnally subperiodic *Wuchereria* *bancrofti* infection in *Downsiomyia* *nivea* (L) after nine rounds of Mass Drug Administration in Nancowry islands, Andaman and Nicobar Islands, India" (#PNTD-D-19-01106)**

****************

**Summary**

Molecular xenomonitoring is a surveillance strategy to detect circulating filarial parasites in an endemic population which is either under intervention or post-intervention. It is an important surveillance tool when human sample collection is difficult. This manuscript provided data from an endemic area where parasite load is high even after many rounds of MDA. The parasite is diurnal in that area where most of the people are outside of their house so blood collection for diagnosis is difficult. Also, they undertook many sampling strategies for the specific vector which will be an important add to the scientific literature and benefit scientists and researchers of similar interest. However, the manuscript has failed to clearly portrait its objective. Their assumption for sample collection is incorrect and lacks proper analysis. Some of the analysis lacks enough data. Description could be made in a better way as the article needed to be improved by rewording.

**Specific Comments**

**Abstract**

Background

Page 2, Line 30: How many rounds of MDA, 12 or nine?

Response: It is nine. The error has been corrected in the revised manuscript. Page 2. Line 30.

**Method**

*Line 38: Not all the collection methods were compared.*

Response: This section has been revised. Since the number of mosquitoes collected was too small to make any statistical analysis., we have deleted all comparisons. Page 2. Lines 34-39

**Results**

*Line 42: So mosquitoes from collected only from MLCs were analysed for the infection?*

Response: Yes, since MLC was the only method which yielded required number of vector mosquitoes. This has been clarified in the revised manuscript. Page 2. Lines 40-44.

**Introduction**

*Page 4, Line 72: Correct the information according to the reference. Also, is there any update epidemiology available?*

Response: Another reference is given for the filariasis situation. World Health Organization. Global programme to eliminate lymphatic filariasis: progress report, 2017. Wkly Epidemiol Rec. 2018;91(44):589-604. PubMed PMID: 28984121. An update is available but not included.

*Line 72: Replace persons by people*

Response: Persons have been replaced by people Lymphatic filariasis (LF), is endemic in 72 countries where about 1.39 billion persons people are at the threat of acquiring infection. Page 5. Line 78.

*Line 77: Replace worldwide by global*

Response: Agreed and corrected. Page 5. Line 80.

India, contributing around 44.3% of the global burden launched the National programme to eliminate LF in 2004, in 256 endemic districts in 21 states and Union territories with about 610 million at risk of infection. Page 5. Line 91-93

*Line 79-80: Are these two drugs used simultaneously?*

Response: Yes, the two drugs were administered simultaneously. Page 5. Lines 93-94.

Administration of MDA with diethylcarbamazine citrate (DEC) and albendazole (ALB) simultaneously is the main strategy to interrupt transmission besides recommending integrated vector management, wherever feasible.

Line 82: Latin name at the beginning of the sentence has to be full.

Response: Agreed. *Wuchereria* *bancrofti* is prevalent in all the endemic states while *B*. *malayi* is restricted to six states and union territories in India. Page 5. Lines 95-97.

*Page 5, Line 110: So DEC-fortified salts are also implemented, or it is the regular mode of intervention? It came all of a sudden. Tell something about the implementation before. What are you evaluating with xenomonitoring, MDA or DEC-salt or both?*

Response: This aspect has been revised and the aim has been clarified in the revised manuscript. The present study was carried out to evaluate the impact of nine rounds of MDA using MX. However, collection of the vector mosquito has been a challenge, though Biogent Sentinel (BGS) is shown to be useful for other Aedes vector species elsewhere. Therefore, apart from BGS, we used different mosquito sampling devices for collection of the vector species in the islands to compare and identify the most productive one. The study also aimed at assessing the vector infection by molecular assay developed for *Culex* *quinquefasciatus* - *W*. *bancrofti* combination, which could be used as a surveillance tool in evaluating MDA in *Ae*. *niveus* transmitted *W*. *bancrofti* infection. Page 5. Line 124-131

*Line 111: Add ‘by xenomonitoring of Do. nivea’ at the end of the sentence?*

Response: Please refer to the above response.

*Line 112: Sampling method, placement, time and selection of region is very crucial for xenomonitoring. Perhaps add this in the introduction. As this manuscript indicated a successful sampling technique like MLC after comparing a number of methods.*

Response: The objectives of the study has been clarified. Please refer the above responses.

**Materials & Method**

Page 6, Line 121: Reference needed.

Response: Reference on the demography has been provided.

*Line 125: Mention the tsunami year.*

Response: Tsunami occurred in 2004 and is given in the revised manuscript. Page 7. Line 146.

*Page 7, Line 142: Explain why the expected prevalence is 1% when it was already more than 1% in the islands.*

Response: Microfilaria survey carried out in 2011 showed an average prevalence of 3%. We assumed a Mf prevalence of 1%, which is the threshold recommended for conducting transmission assessment survey (TAS) for arriving at a decision on stopping MDA. Expecting random biting of mosquitoes at this prevalence level in human, we assumed prevalence of infection in mosquito at 1%. The sample size derived was 1592 mosquitoes from the four islands, with an absolute precision of 0.5% (0.5-1.5%) and a design effect of 1 at 95% confidence level.

*Line 150: The trapping sites mentioned here are already mentioned the earlier paragraph. Again, how many islands actually? Previously you have mentioned three out of four but in here it is three out of five?*

Response: The collection (trapping) sites are given in the beginning of collection methods and the repetition has been removed. Trap collections were conducted in 21 villages in 4 islands. MLCs were conducted in all the 25 villages in five islands. This has been clearly mentioned in the revised manuscript. Page 8. Line 160-166.

Page 9, Line 186: This is totally unnecessary. Desiccants keep the mosquitoes good enough for PCR.

Response: The mosquitoes were dried overnight. Alternatively, it can be dried at 95^0^C for 10 minutes

Page 11, Line 214: What is the timeline of the study?

Response: The entire study was undertaken during the period between May 2014 and July 2015. The collections using the three traps were conducted between May 2014 and December 2014. MLCs were conducted between December 2014 and July 2015. These details have been provided in the revised manuscript. The dates for mosquito collections using traps and MLC are provided in the revised manuscript. Page 10. Line 207. Page 10. Line 216. Page 11. Line 240. Page 11. Line 243.

Line 218: You have selected volunteers from the endemic region. Did you check if the volunteer already have the infection? How many volunteers have been used and how many MLCs were conducted? What is the follow-up process of the volunteers? Any benefit given to them?

Response: A total of 151 MLCs were conducted in 77 collection sites. Volunteers were selected from each village and were used in all the collections conducted in a given village and collection site. These volunteers were covered under m*f* survey in these villages and were free from *mf*. Expenses for the refreshments provided for the period of collection were met. These volunteers were included in the follow-up *mf* prevalence surveys in these villages

Page 12, Data analysis, Line 253-255: Have to be careful with count data. Table 2 indicates that the data are over dispersed. Mean is not the proper way to compare between locations or traps. How did you measure the densities? Need better comparison tool like risk ratio for collection location. How did you calculate man landing rate?

Response: Tables 2 & 3 have been deleted and analysis aspect has been deleted considering the small number of mosquitoes obtained from different traps.

*Additional Comment: Can you provide a map of the islands with collection sites according to methods?*

Response: Map of the islands depicting collection sites according to methods is provided.

**Results**

*Page 14: Line 273: Use the term ‘BGS’ uniformly*

Response: The term ‘BGS” has been uniformly used in the revised manuscript.

*Line 281: Sometime mean and standard deviation is used and sometime not used. Be uniform throughout the document.*

Response: Please refer to the response provided for Q.12

*Page 15, Line 285: What are these?*

Response: The results of statistical analysis. This has been removed.

*Page 17, Table 3: The sample size is too low for any kind of comparison. Table 3 should be taken out.*

Response: Agreed. It has been deleted.

*Page 18, Line 300: Figure number missing*

Response: The Figure number 1 has been given.

*Line 298-300 and 304-306: The list of mosquitoes are not required. Just mentioned one or two dominant species collected from these traps.*

Response: The number of not only the vector species but also other mosquitoes. The list will be useful to the readers to understand the mosquito profile in the island situations and distribution of mosquitoes in the country.

*Line 306-308: Re-write the sentence like: The mean number of mosquitoes collected per trap ranged between 0.07 to 0.38 in collection sites.*

Response: This aspect has been deleted in view of less numbers of mosquitoes collected.

*Line 309: Provide a figure number even if there is no more figure. DBNT or HBDNT?*

Response: The figure number has been provided.

*Line 317: Why it is density?*

Response: It has been corrected as man landing rate in the revised manuscript.

*Line 318-323: Be uniform providing mean and standard deviation*

Response: Since we have deleted this aspect of comparisons in view of the small numbers of mosquitoes, mean and standard deviation also has been deleted.

*319: ‘96.94’ is it Kamorta or Nancowry?*

Response: It is Nancowry. This has been corrected in the Table 4.

*Page 20, Table 4: Calculation of man landing rate and per man hour density is not clear. Provide description in the data analysis.*

Response: This has been provided in the methodology section.

*Page 21, Line 331: Give % after 7.6*

Response: % after 7.6 has been given

*Line 332: Same as previous*

Response: % is given after the figure

Line 337: Font not same, give 5 after 0.43

Response: The font has been made uniform. % has been provided after 0.43

**Discussion**

Page 23, Line 352: Any possible reason behind the failure of so many MDA rounds?

Response: Could be community compliance

*Line 359: What is XM?*

Response: Mx is xenomonitoring-it was an error and corrected.

*Line 359-361: Proper message from this sentence is missing. Mf prevalence >1% would provide better yield for surveillance by methods like xenomonitoring when it is difficult sample human blood.*

Response: The paragraph has been revised.

Line 367: Are these supposed to be productive when the mosquito is forest dwelling?

Response: It may be true for the gravid traps. But other collections target host seeking mosquitoes. This has been revised.

*Line 368: Anything missing from this sentence?*

Response: This paragraph has been revised in response to the comments of other reviewers.

*Line 370: Delete ‘to’*

Response: “to” is deleted in this sentence.

Page 24, Line 372-383: Re-write the whole paragraph. Surprised to see 10 per man hour rate in discussion when not mentioned the results. Why do a sample of 20 is required? Perhaps some more interesting discussion for success of MLC over HBDNT? What is the link of discussing on BGS here with this study?

Response: The entire discussion has been revised. Including the possible reason for the difference in the collection between HBDNT and MLC. Page 17. Line 380-385

Line 385-394: What is the context of discussing *Ae*. *polynesiensis* and BGS? Delete this paragraph or reword.

Response: *Ae*. *polynesiensis* is also a vector and day biting mosquito. BGS trap was shown to be productive in sampling this mosquito species. In this context it was discussed and has been revised. Page 17. Line 393-396

*Page 25, Line 396-398: Merge the two sentences and a better rephrasing needed.*

Response: Rephrased the sentence as follows:

Vector infection was detected in all the four islands, indicating the source of infection in the community. Also, the upper CI of vector infection was above the threshold of 0.1% (Ref. WHO 1996) in all the islands and hence are at the risk of continuing transmission.

Line 398-400: This is an important finding supporting molecular xenomonitoring as a surveillance strategy for filariasis. Perhaps add some more evidence here. Bring the next paragraph here.

Response: As suggested the paragraph has been moved.

Line 406-411: There is repetition from the expression of present study outcome, merge the two sentences.

Response: This paragraph has now been revised in line with the comments of another reviewer.

Page 26, Line 426: Give evidences from other countries

Response: MX can be a potential tool to assess DspWB during post- intervention period as has been reported for nocturnally periodic *Wuchereria* *bancrofti* – *Cx*. *quinquefasciatus* (Subramanian et al., 2020, Rao et al (2017) and nocturnally period W. *bancrofti* – An. gambiae

1. Rao RU, Nagodavithana KC, Samarasekera SD, Wijegunawardana AD, Premakumara WD, Perera SN, et al. A comprehensive assessment of lymphatic filariasis in Sri Lanka six years after cessation of mass drug administration. PLoS Negl Trop Dis. 2014;8(11):e3281. Epub 2014/11/14. doi: 10.1371/journal.pntd.0003281.PNTD-D-14-00651 [pii]. PubMed PMID: 25393404; PubMed Central PMCID: PMC4230885.
2. Sheel M, Sheridan S, Gass K, Won K, Fuimaono S, Kirk M, et al. Identifying residual transmission of lymphatic filariasis after mass drug administration: Comparing school-based versus community-based surveillance - American Samoa, 2016. PLoS Negl Trop Dis. 2018;12(7):e0006583. Epub 2018/07/17. doi: 10.1371/journal.pntd.0006583. PubMed PMID: 30011276; PubMed Central PMCID: PMCPMC6062125.
3. Lau CL, Won KY, Becker L, Soares Magalhaes RJ, Fuimaono S, Melrose W, et al. Seroprevalence and spatial epidemiology of Lymphatic Filariasis inAmerican Samoa after successful mass drug administration. PLoS Negl Trop Dis. 2014;8(11):e3297. Epub 2014/11/14. doi: 10.1371/journal.pntd.0003297. PNTD-D-14-01061 [pii]. PubMed PMID: 25393716; PubMed Central PMCID: PMC4230933.
4. Schmaedick MA, Koppel AL, Pilotte N, Torres M, Williams SA, Dobson SL, et al. Molecular xenomonitoring using mosquitoes to map lymphatic filariasis after mass drug administration in American Samoa. PLoS Negl Trop Dis. 2014;8(8):e3087. Epub 2014/08/15. doi: 10.1371/journal.pntd.0003087.PNTD-D-14-00593 [pii]. PubMed PMID: 25122037; PubMed Central PMCID: PMC4133231

**Conclusion**

Page 26, Line 431: There are already many methods alternative to MLC but it’s just the species specific in this case.

Response: Agreed. Conclusion section has been modified. Page 19. Line 437

Line 432-434: Not necessary.

Response: This sentence is important as it is one of the outcomes of the study.

Line 434-436: As commented before, it is not clear under what circumstance MX is potential. Is it for MDA, DEC-salt or both?

Response: MX in *Ae*. *niveus* can be a supplementary to TAS and post-MDA period for early detection of risk of transmission. Page 19. Line 441-442

**References**

Check with the journal’s format guidelines. Doesn’t look like the style is followed properly.
